# Engineering polymer MEMS using combined microfluidic pervaporation and micro-molding

**DOI:** 10.1038/s41378-018-0017-2

**Published:** 2018-07-02

**Authors:** Damien Thuau, Cédric Laval, Isabelle Dufour, Philippe Poulin, Cédric Ayela, Jean-Baptiste Salmon

**Affiliations:** 1 Laboratoire IMS, University of Bordeaux, UMR 5218, ENSCBP, 16 avenue Pey Berland, 33607 Pessac, France; 2 Centre National de Recherche Scientifique, University of Bordeaux, Solvay, LOF, UMR 5258, 33600 Pessac, France; 3 Centre de Recherche Paul Pascal, University of Bordeaux, Avenue Schweitzer, 33600 Pessac, France

## Abstract

In view of the extensive increase of flexible devices and wearable electronics, the development of polymer micro-electro-mechanical systems (MEMS) is becoming more and more important since their potential to meet the multiple needs for sensing applications in flexible electronics is now clearly established. Nevertheless, polymer micromachining for MEMS applications is not yet as mature as its silicon counterpart, and innovative microfabrication techniques are still expected. We show in the present work an emerging and versatile microfabrication method to produce arbitrary organic, spatially resolved *multilayer* micro-structures, starting from dilute inks, and with possibly a large choice of materials. This approach consists in extending classical microfluidic pervaporation combined with MIcro-Molding In Capillaries. To illustrate the potential of this technique, bilayer polymer double-clamped resonators with integrated piezoresistive readout have been fabricated, characterized, and applied to humidity sensing. The present work opens new opportunities for the conception and integration of polymers in MEMS.

## MEMS: Versatile technique paves the way for new flexible devices

An innovative and versatile technique for fabricating micro electromechanical systems (MEMS) from polymers opens the door to new sensing technologies for use in flexible devices. Owing to their low cost, flexibility, and biocompatibility, polymer and composite materials are attracting considerable attention as an alternative to silicon-based MEMS for biological applications, and energy and sensing technologies. Current manufacturing processes for polymer-based MEMS, however, still lag behind those for silicon. Now, Damien Thuau and colleagues from the University of Bordeaux in France have used classical microfluidic pervaporation combined with micro-molding to produce multilayer polymeric micro-structures. The technique could be used for creating micro-structuration from almost any type of materials, starting from dilute colloidal dispersions to polymer solutions, and paves the way for new applications, including bio-sensors, mechanical energy harvesters, and actuators

## Introduction

Micro-electro-mechanical systems (MEMS) were made of silicon (Si) material for a long time using fabrication technologies derived from the semiconductor integrated circuit industry. Recent advances in materials science and mechanical engineering have introduced the use of polymers in MEMS devices to meet the increasing needs of future applications^1,2^. Owing to their particular properties (low cost, flexibility, biocompatibility), polymer and composite material-based MEMS have the potential to be a powerful alternative to Si-based MEMS devices for their use in biological applications, mechanical energy harvesting, or for any other application requiring sensing and actuation^3^. While Si-based microfabrication techniques are well established and understood, new processing techniques still need to be developed for engineering organic microscale and nanoscale materials^4^. Polymer micromachining has originally focused on organic materials commonly used in microelectronics (photosensitive epoxy (SU-8), polyimide, parylene, acrylics (PMMA), and poly(dimethylsiloxane) (PDMS)) processed by standard micromachining techniques^5–8^. In particular, photolithography offers mature equipment and expertise, and thus high-throughput microfabrication. In order to broaden the limited choice of polymers compatible with photolithography, different approaches have been developed over the past years for the conception of polymer micro-structures^9–17^. 2D and 3D microfabrication processes with a resolution of 10 µm were reported by combining classical deposition and release techniques with a numerical control cutting plotter^9^. Various devices including temperature or viscosity sensors based on resonant micro-structures with integrated actuation and readout schemes were fabricated, proving the capability of this technique for rapid, simple, and environmentally sustainable conceptions of microsystems. To overcome the limitations of solution-based processes, shadow masking is becoming a standard technique for patterning low-molecular-weight materials (e.g., organic semiconductors for active layers in organic light-emitting diodes and organic thin-film transistors for instance) which are usually processed into films using vacuum deposition^10^. Nevertheless, the size of the shadow masks and the required alignment steps for integrating functional MEMS still limit the downscaling of such devices and their performances. Hot embossing^11,12^ and nanoimprint lithography^13^ were also employed in microscale and nanoscale processing of soft materials for sensing applications. Although facing reliability and repeatability issues, these approaches led to promising thermal actuators and polymer accelerometers. Inkjet printing, on the other hand, is of particular interest to pattern polymeric materials that are processed into films starting from inks^14,15^. Among these numerous techniques, however, there is little work regarding the development of *free-standing multilayer* polymer micro-structures, such as MEMS resonators and actuators^16,17^. Indeed, the conception of polymer MEMS containing multiple layers, that is, passive (structural, encapsulation) and/or functional (electromechanical transducer, sensitive or functional layers), remains challenging for many printing technologies, mainly owing to the complex interplay between a large number of process parameters like ink rheology, surface tension effects, drying kinetics, solvent interactions, and the need of sacrificial layers.

**Fig. 1:**
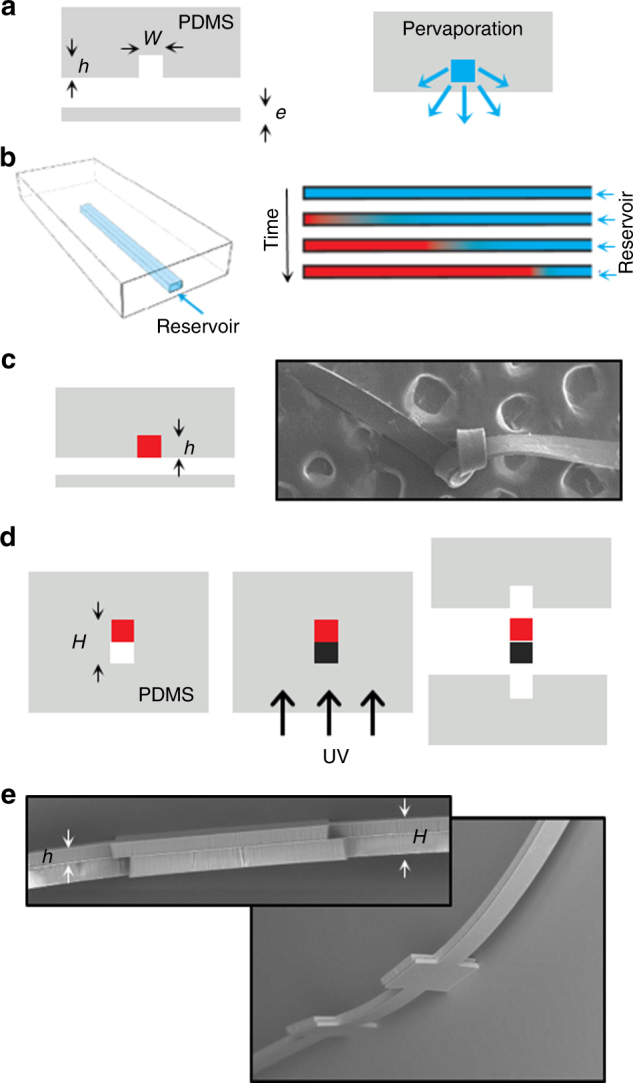
Fabrication process flow of multilayer polymer micro-structures. **a** (Left) Reversible sealing of dead-end channels embedded in a PDMS mold by a PDMS membrane. Typical dimensions are *h* *=* 10–50 µm, *e* *=* 10–50 µm, and *w* *=* 50–1000 µm. (Right) Transverse view evidencing the solvent pervaporation flux through the PDMS membrane when the channel is filled by a dilute ink (blue). **b** (Left) schematic 3D view of the pervaporation-induced flow for a single channel (blue arrow). (Right) corresponding schematic pervaporation-induced growth of a material (red). **c** (Left) removal of the PDMS stamp from the membrane. (Right) the resulting micro-materials are rigid enough to be handled manually, as shown by the SEM image of a simple PVA-CNT beam in which we made a knot (width 150 µm, thickness *h* = 30 µm). **d** Alignment of a second structured PDMS mold with the material still embedded in the first block (red). Filling of the channel using a photo-curable polymer (black) makes it possible to fabricate a second layer after UV polymerization (MIMIC process). **e** (Left) SEM transverse view of an NOA81/PVA-CNT composite bilayer, the total thickness of the structure is *H* = 115 µm. (Right) Perspective SEM view of the same micro-structure, the width of the main channel is 150 µm

The MIcro-Molding In Capillaries (MIMIC) approach developed by Xia and Whitesides^18^. MIMIC could be an alternative route to overcome such difficulties. This classical technique makes possible the fabrication of organic micro-materials with complex shapes in one step, starting from pre-polymer solutions injected within a micro-structured PDMS stamp. Our group extended this approach by combining the solvent *evaporation* through the PDMS matrix, and more precisely through a thin PDMS membrane, to allow the fabrication of micro-materials starting from any *dilute* inks. Many solvents, and in particular water, are indeed known to permeate through PDMS by a mechanism known as *pervaporation*, combining the solubilization of the solvent molecules within the PDMS membrane, their diffusion up to the side exposed to air, and their further evaporation^19^. Figure 1a briefly illustrates the *microfluidic pervaporation* technique for making micro-materials starting from dilute inks. Dead-end channels of arbitrary shapes (2D or 2 + 1D) are first made using soft lithography in a PDMS block, sealed by a thin PDMS membrane (typical thickness ranging from 10 to 50 µm). Such channels are connected to a reservoir containing a dilute ink, and filled by the latter. Water pervaporation, mainly through the PDMS membrane, drives in turn a flow from the reservoir up to the channel tip owing to mass conservation (see Fig. 1b for the case of a single channel). The pervaporation-induced flow rate typically ranges from 0.01 to 1 µl h^−1^ for a single channel, depending on the geometrical features of the channels, the membrane thickness, and the external humidity^20,21^. This flow consequently enriches continuously the tip of the channel with non-volatile species contained in the reservoir, up to the formation of a solid material that eventually invades the channel (see Fig. 1b)^22^. Over the past few years, we investigated in depth these mechanisms, mainly for aqueous dispersions of nanoparticles^23^ and demonstrated successful fabrications of nanoparticle-dense assemblies, with applications ranging from optical metamaterials^24^ to surface-enhanced Raman spectroscopy substrates^25^. Demko et al.^26,27^ also reported similar works and extended the pervaporation technique to the use of non-aqueous dispersions, using the development of a solvent-compatible permeable matrix.

In a recent work^22^, we performed a detailed investigation of the opportunities offered by this technique to obtain composite micro-materials starting from dilute polymer solutions containing colloidal species. More precisely, we investigated the case of carbon nanotubes (CNTs) dispersed in polyvinyl alcohol (PVA) aqueous solutions^28–30^. Microfluidic pervaporation again makes possible the growth of a wide variety of composite micro-structures, see for instance Fig. 1c showing the case of a simple beam in which we made a knot, or Figure S1 for complex micro-structures including 2 + 1D beams, honeycombed networks, and other complex mechanical structures manually handled. The growth kinetics of such polymeric materials can be quantitatively predicted by simple mass conservation equations owing to the outstanding control of transport phenomena at the microfluidic scale. In particular, the typical growth time of a composite material only depends on the transverse dimensions of the channel (height × width), the pervaporation rate in the case of pure water, and the concentration of the dilute ink^22^. We also showed that the transverse dimensions of the final micro-structures are slightly smaller than the targeted dimensions imposed by the PDMS mold, owing to the shrinkage of the material during the pervaporation-induced solidification of the ink^22^. These phenomena result from a subtle coupling between the build-up of mechanical stresses during solidification, mechanical deformations of the PDMS matrix, and adhesion between the composite and the PDMS walls. Demko et al.^26,27^ showed that the use of a more rigid, but still permeable, matrix allows to minimize such deformations.

Although microfluidic pervaporation has been largely reported as an efficient and versatile technique to fabricate micro-scaled structures with complex shapes, there is no work, up to date, reporting the fabrication of polymeric functional *free-standing multilayer* micro-structures, which constitute, however, important building blocks for MEMS resonators and actuators. This challenging task is the objective of this work. In this regard, we report below an original multi-step process with alignment procedures to extend this technique to the fabrication of multilayer polymeric micro-structures. As a proof of concept, we also demonstrate the successful fabrication of a bilayer polymer double-clamped resonator with an integrated piezoresistive readout for humidity sensing.

## Materials and methods

### Formulations of PVA-CNT inks

A stock aqueous solution of PVA (72 kg mol^−1^, Sigma-Aldrich) was prepared using deionized water at a mass fraction of 7.5%. To solubilize PVA, the solution is heated at 90 °C for 1 h and left to cool to room temperature (12 h). We also prepared a 10 g stock aqueous CNT dispersion by sonicating 90 mg of multi-walled CNT (Arkema, GraphiStrength C100) with 120 mg of Brij78 (polyoxyethylene glycol alkyl ether surfactant, Sigma-Aldrich). Inks investigated in the present work were obtained through appropriate mixing and dilutions of the two above stock solutions. Initial volume fractions of PVA are in the range 0.001–0.03 (assuming the PVA density 1.27 g cm^−3^). CNT mass concentration in the composite material is estimated assuming the additivity of the volumes and the relative density of CNT to 1.8.

### Fabrication of the microfluidic chips

We used standard photolithography and alignment processes to make networks of dead-end channels with different heights on a silicon wafer (photoresist SU-8). We then used standard soft lithography techniques to obtain networks of channels embedded within a PDMS matrix (Sylgard 184, Dow Corning, curing agent ratio 1/10, typical curing time 1 h at 65 °C). To seal reversibly PDMS channels with a PDMS membrane, as shown in Figure 1a, we proceeded as follows. We first spin coated a thin PDMS layer (typical thicknesses 10–50 µm) on a silicon wafer, and we cured it at 65 °C for about 1 h. A thicker PDMS block (~ 1 cm) is also reticulated on another silicon wafer, carefully peeled off, and cut to get a square opening (2 cm × 2 cm) in it. This thicker PDMS block is then plasma bonded on the thin PDMS layer obtained by spin coating. The final device is again peeled off from the wafer to get a thin PDMS membrane supported by the thicker stamp for mechanical strength. The micro-structured PDMS stamp containing the channels is finally peeled off from the wafer, punched to connect tubes, and carefully deposited on the membrane (see Fig. 1a). Natural adhesion between the PDMS membrane and the PDMS micro-structured stamp makes it possible to fill the dead-end channel networks without leakages.

### Characterization of MEMS resonators

**Fig. 2:**
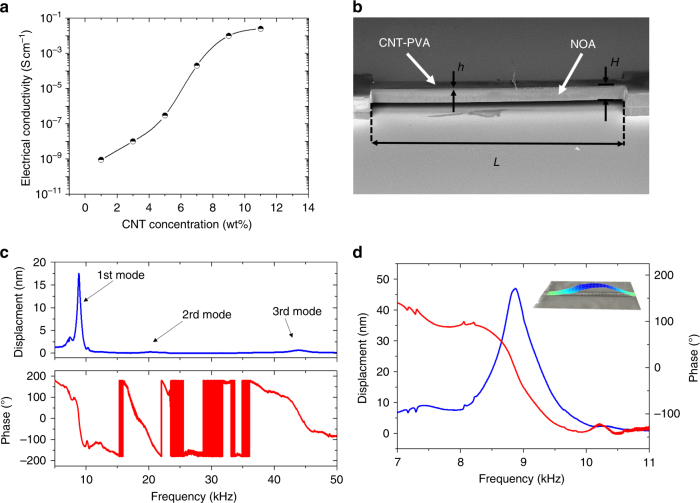
**a** Electrical conductivity of PVA-CNT composites as a function of CNT concentration. **b** SEM image of a double-clamped piezoresistive polymer resonator made by the process illustrated in Figure 1, *H* = 115 µm, *h* = 20 µm. **c** Vibration amplitude and phase of the first, second, and third flexural out-of-plane modes of resonance measured optically. **d** Zoom on the magnitude of displacement and phase of the first flexural out-of-plane resonant frequency measured optically. Inset: snapshot of the mode shape of the first resonance mode measured optically

The composite micro-materials are rigid enough to be handled manually or using precise tweezers. Some of these micro-materials were deposited on metallic pins covered by a conductive tape to obtain scanning electron microscopic (SEM) images using a tabletop SEM (Hitachi TM3030). The fabricated bridge shown in Figure 2b was driven into resonance by an external piezoelectric actuator. The dynamic behavior of the polymer resonators was characterized optically using a laser Doppler vibrometer MSA 500 from Polytec and electrically using a network analyzer (Agilent E5061B). All measurements were performed in air at atmospheric pressure. Humidity monitoring was performed using a vapor generator instrument from Surface Measurement Instrument Ltd where temperature was monitored using a Eurotherm 2604 temperature controller .

## Results and discussion

To illustrate the capabilities of the pervaporation technique for the patterning of multilayer micro-structures with applications in the field of integrated polymer MEMS, we fabricated bilayer resonators with integrated piezoresistive electromechanical transduction. The fabrication process flow is illustrated in Figure 1. The first step consists in the fabrication of a piezoresistive functional layer using microfluidic pervaporation starting from dilute CNT-PVA inks, as already reported in ref.^22^. The growth kinetics typically range between 1 to 20 h for the experimental range of concentration investigated. This technique can be used to make a wide variety of structures, typically with final transverse dimensions *h* ranging between 10 and 100 µm, and widths up to 1 mm, see the SEM images in Figure S1 for some examples. Importantly, despite the unavoidable slight shrinkage of the targeted dimensions owing to the generation of stresses during the solidification of the ink (typical shrinkage ratio of the transverse dimensions ranging from 0.4 to 0.8)^22^, the final material features are highly reproducible owing to the fine control of the growth kinetics of such materials imposed by the microfluidic scale.

The second step consists in the fabrication of a structural layer on this polymer micro-structure. After the complete growth of the composite material (Fig. 1d), the PDMS stamp is carefully detached from the membrane and aligned with a second PDMS block containing microfluidic channels, with possibly 2 + 1D shapes (Fig. 1d). Alignment of the two blocks is performed under a standard mask aligner. This second block is then used to pattern the second layer by the MIMIC process, by gently filling the channels with, for instance, ultraviolet (UV)-photo-curable polymers (e.g., Norland Optical Adhesive NOA81 or formulations of polyethylene glycol diacrylate + photoinitiators such as 2,2-dimethoxy-2-phenylacetophenone). Further UV exposure reticulates the resist leading to the formation of a solid layer firmly bonded to the functional layer obtained by pervaporation. After the complete fabrication of the composite bilayer material, PDMS stamps are again carefully removed, and the soft adhesion between the two PDMS surfaces prevents from imposing mechanical strains to the materials. Figure 1e show SEM images demonstrating the successful fabrication of such bilayer polymer micro-materials.

To go a step further into the demonstration of the opportunities offered by this technique, we fabricate the rectangular suspended bridge resonator shown in Figure 2b, and we demonstrate the piezoresistive readout of the strain induced into the resonator during vibration. This micro-structure was obtained using the process diagram shown in Figure 1, and is made of a functional PVA-CNT layer of thickness *h* = 20 µm bonded to a NOA81 2 + 1D structural layer including the pillars. The whole dimensions of the resonator are *L* *=* 4000 µm in length, 150 µm in width, and *H* *=* 115 µm in thickness (excluding the pillars). The piezoresistive transduction mechanism of the functional layer relies upon the resistivity variation of the CNT-PVA material under a mechanical strain. The piezoresistive behavior of a CNT network dispersed in an insulating PVA matrix involves a complicated interplay among different mechanisms, including the resistance change of the composite due to the dimensional changes, tunneling resistance of the CNTs, resistance of the intertube contacts, and intrinsic piezoresistivity of the nanotubes. CNT-based composites show an enhancement of the piezoresistive sensitivity for a CNT concentration in the percolation region^6^. To estimate the percolation concentration of the PVA-CNT composite under study, the electrical conductivity of several PVA-CNT planar beams of 1 cm long, 100 µm wide, and 25 µm high obtained from microfluidic pervaporation were characterized. The electrical conductivity of the different PVA-CNT composite structures containing a CNT concentration ranging from 1 to 11 wt% were measured using a 4-point measurement setup PRO4 from Microworld connected to a Keithley 2400 Sourcemeter. As shown in Figure 2a, the electrical conductivity of the material increases by about 8 orders of magnitude as the CNT concentration increases from 0 to 11 wt%. Such results are in line with the existence of a percolation threshold above which CNTs form a conductive path in the polymer matrix, causing the composite to change from being insulating to conductive. As a matter of fact, the electrical conductivity shows an abrupt increase, from 1 × 10^−8^ to 1 × 10^−2^ S cm^−1^ for composites with a CNT concentration ranging between 4 and 9 wt%, defined as the percolation region. In the following, we choose PVA-CNT composite with a CNT concentration of 8 wt% for the piezoresistive functional layer of the NOA resonator shown in Figure 2b, as the latter is expected to display the highest sensitivity to strain.

MEMS can operate either in a static bending regime or in dynamic mode. In the latter, the phenomenon to be sensed may be revealed with excellent resolution by a shift in the resonant frequency of the resonator caused by, for example, sorbed mass and/or environmentally induced changes in material properties. Sensing based on resonant frequency shift provides more precision than sensing in the static regime since the effect of noise in a direct voltage or current measurement is clearly attenuated in frequency measurements. To this end, the fabricated polymer MEMS were driven into resonance by using an external piezoelectric actuator while the vibration amplitudes and phases of the first, second, and third flexural out-of-plane modes of resonance were measured optically (see Fig. 2c, d). These experimentally measured values were compared to analytical resonant frequencies (*f*_r_) obtained from refs. ^31,32^

$$f_{\mathrm{r}} = \frac{{\lambda ^2H}}{{2\pi L^2}}\sqrt {\frac{E}{{12\rho }},}$$

where *λ* is a dimensionless parameter which is a function of the boundary conditions applied to the beam, and in the case of clamped–clamped beam, it is equal to 4.73, 7.85, and 11.00 for the first, second, and third mode of resonant frequency, respectively, *H* is the beam thickness, *L* its length, *E* its Young’s modulus estimated at 1.30 GPa, and *ρ* the density (1230 kg m^−3^). As shown in Table 1, a relatively good agreement between analytical and experimental values was obtained for the given geometrical dimensions and physical properties used in the calculations. The observed discrepancy of <10% between the analytical values and the experimental data were assumed to arise from the non-ideal clamping during the mounting process, leading to a less stiff bridge actuator behavior. Such differences could be reduced by using wafer-bonding processes improving the adhesion between the resonator and the base^33^.

**Table 1:** Comparison between the calculated resonant frequencies of the first, second, and third out-of-plane modes, and the experimental optical measurements

	Resonant frequency (*f*_r_)		
	Analytical (Hz)	Experimental (Hz)	Error (%)
1st mode	8160	8850	8.5
2nd mode	22,500	20,260	9.9
3rd mode	44,100	43,850	0.5

**Fig. 3:**
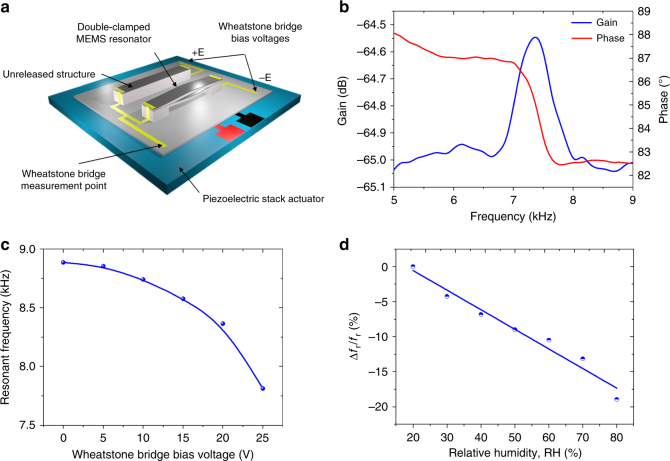
**a** Schematic view of the half Wheatstone bridge configuration of the piezoresistive transduction. **b** Piezoresistive detection of the first flexural out-of-plane resonance mode. **c** Variation of resonant frequency of the first flexural out-of-plane mode measured optically with a laser vibrometer as a function of Joule heating resulting from an applied bias voltage to the bridge resonator. **d** Relative change of the resonant frequency as a function of relative humidity RH

The piezoresistive detection of the resonator was achieved by recording the resistance change of the double-clamped resonator arranged in a half Wheatstone bridge configuration without using any amplification. As shown in Figure 3a, the actual chip under study is composed of a released resonator for the measurements, and another unreleased was used as a reference for differential piezoresistive detection. The reference piezoresistive bridge has the same electrical environment and identical geometrical dimensions as the suspended resonator. This reference micro-structure and the vibrating bridge were fabricated in similar identical conditions starting from the same dilute ink using a single microfluidic pervaporation experiment, but with a specific PDMS mold to obtain two different passive layers, bridge pillars vs. filled structure. The resonant frequency of the first out-of-plane flexural mode obtained with the piezoresistive readout was measured at 7560 Hz (Fig. 3b) and compared with the value obtained optically at 8850 Hz (Fig. 2d). The decrease of the resonant frequency measured electrically comes from the softening of the materials composing the MEMS resonators by Joule heating due to the bias voltage (±25 V) applied to the bridge during the piezoresistive detection. As expected, the resonant frequency, when increasing the bias voltage, showed a shift towards lower values (see Fig. 3c). More precisely, when a bias voltage of ±25 V was applied to the resonator, a relative shift of the resonant frequency of 13% towards lower values was measured optically, in agreement with the resonant frequency obtained electrically, by the piezoresistive detection scheme.

Since humidity is expected to slightly soften the PVA matrix, the bilayer CNT-PVA/NOA resonator has been tested for humidity sensing applications as a proof of concept. The dynamic electromechanical response of the integrated polymer double-clamped resonators as a function of the ambient relative humidity (RH) has been monitored. The measured relative changes of resonant frequency (Δ*f*_r_*/f*_r_) vs. humidity are plotted in Figure 3d. A large drop of 17% of the resonant frequency (from 7899 to 6402 Hz) when RH increases from 20 to 80% has been observed. Moreover, the integrated polymer MEMS resonators present excellent linear response, reversibility, and repeatability after cyclic measurements in this humidity range. The sensitivity of the PVA/CNT-based resonator has been determined from a linear regression to be −2800 ppm %RH^−1^ in the range 20 to 80%RH. Such a large sensitivity makes the proposed resonators some of the most sensitive polymer MEMS-based humidity sensor reported to date^34^. In a broader context, the large choice of materials available with adjustable mechanical properties associated with countless devices’ geometries highlights the potential of microfluidic pervaporation for the conception of advanced polymer microsystems.

## Conclusion

By contrast to conventional microfabrication processes such as micro-molding or photolithography that are limited to UV-curable or heat-curable polymers or thermoplastics^35–38^, microfluidic pervaporation combined with MIMIC a priori allows the micro-structuration of almost any type of materials starting from dilute colloidal dispersions to polymer solutions. While previously reported microfluidic pervaporation studies allowed structuring single layers of polymeric materials (see e.g., ^22^), the multi-step process reported in the present work now makes it possible to design advanced polymer MEMS with complex architectures and bilayer structures. Importantly, the unavoidable build-up of mechanical stresses during the pervaporation-induced solidification of the ink, and/or during the UV photo-polymerization of the second layer, may lead to a slight shrinkage of the actual dimensions of the structures (as compared to the targeted ones), and/or to trapped internal stresses. Such issues are beyond the scope of the present work, as they would require dedicated studies, but are nevertheless fundamental for further applications of our technique with a wider range of polymeric inks.

Mastering the fabrication of multilayer micro-structures offers the possibility of proposing integrated readout schemes that in turn increases integration and application possibilities of the first generation of single layer devices. This fabrication method can be applied to a large panel of organic materials opening new routes towards the development of polymer MEMS. In particular, polymeric piezoresistive resonators have been developed and characterized, demonstrating their capability to be used as promising actuators and sensors. The piezoresistive polymer MEMS resonators were also characterized optically and electrically. As a proof of concept, they were tested as humidity sensors exhibiting a remarkable sensitivity of −2800 ppm %RH^−1^.

Moreover, we showed recently that microfluidic pervaporation combined with actuated micro-valves makes it also possible to even design micro-materials with any programmed composition gradient, a unique feature compared to classical microfabrication techniques^39^. Considering the cost, the versatility, the performance, and the opportunities offered by the proposed approach, microfabrication of polymeric MEMS using microfluidic pervaporation offers exciting new possibilities across a wide spectrum of applications, including bio-sensors, mechanical energy harvesters, and actuators.

## Electronic supplementary material

Figure S1
