# Supplementary material for: Engineering polymer MEMS using combined microfluidic pervaporation and micro-molding
Source: Microsyst Nanoeng. 2018 Jul 2;4:15. doi: 10.1038/s41378-018-0017-2 (PMC6220165; doi:10.1038/s41378-018-0017-2)
Supplement: Supplementary file 1 — Figure S1 [file 41378_2018_17_MOESM1_ESM.docx]

**Supplementary information**

Engineering Polymer MEMS using combined Microfluidic Pervaporation and Micro-Moulding

*D. Thuau^1^, C. Laval^2^, I. Dufour^1^, P. Poulin^3^, C. Ayela^1^, J-B. Salmon^2^*

*^1^ Univ. Bordeaux, Laboratoire IMS, UMR 5218, ENSCBP, 16 avenue Pey Berland, 33607, Pessac Cedex, France*

*^2^ Univ. Bordeaux, CNRS, Solvay, LOF, UMR 5258, F-33600 Pessac, France*

*^3^ Univ. Bordeaux, Centre de Recherche Paul Pascal, Avenue Schweitzer, 33600 Pessac, France*

Figure S1. SEM images of composite polymer microstructures made by microfluidic pervaporation, starting from dilute aqueous inks PVA-CNT. (a) height of the structure 30 µm; (b) width of the shoelace 100 µm; (c-e) channels width 40 µm. In (b), the shoe has been laced up manually starting from a planar microstructure.
